# Substitution Mapping of Two Closely Linked QTLs on Chromosome 8 Controlling Grain Chalkiness in Rice

**DOI:** 10.1186/s12284-021-00526-4

**Published:** 2021-10-02

**Authors:** Weifeng Yang, Liang Xiong, Jiayan Liang, Qingwen Hao, Xin Luan, Quanya Tan, Shiwan Lin, Haitao Zhu, Guifu Liu, Zupei Liu, Suhong Bu, Shaokui Wang, Guiquan Zhang

**Affiliations:** Guangdong Provincial Key Laboratory of Plant Molecular Breeding, State Key Laboratory for Conservation and Utilization of Subtropical Agro-Bioresources, South China Agricultural University, Guangzhou, 510642 China

**Keywords:** Grain chalkiness, Grain shape, Quantitative trait locus, Heat stress, Substitution mapping, Rice

## Abstract

Rice varieties are required to have high yield and good grain quality. Grain chalkiness and grain shape are two important traits of rice grain quality. Low chalkiness slender grains are preferred by most rice consumers. Here, we dissected two closely linked quantitative trait loci (QTLs) controlling grain chalkiness and grain shape on rice chromosome 8 by substitution mapping. Two closely linked QTLs controlling grain chalkiness and grain shape were identified using single-segment substitution lines (SSSLs). The two QTLs were then dissected on rice chromosome 8 by secondary substitution mapping. *qPGC8.1* was located in an interval of 1382.6 kb and *qPGC8.2* was mapped in a 2057.1 kb region. The maximum distance of the two QTLs was 4.37 Mb and the space distance of two QTL intervals was 0.72 Mb. *qPGC8.1* controlled grain chalkiness and grain width. *qPGC8.2* was responsible for grain chalkiness, grain length and width. The additive effects of *qPGC8.1* and *qPGC8.2* on grain chalkiness were not affected by higher temperature. Two closely linked QTLs *qPGC8.1* and *qPGC8.2* were dissected on rice chromosome 8. They controlled the phenotypes of grain chalkiness and grain shape. The two QTLs were insensitive to higher temperature.

**Supplementary Information:**

The online version contains supplementary material available at 10.1186/s12284-021-00526-4.

## Background

Rice is an important food crop in the world. Rice varieties with higher head rice yield, higher transparency and less chalkiness are more popular in the market (Sreenivasulu et al. 2015; Misra et al. 2019). With the increase of living standard, rice varieties are required to have both a higher grain yield and a better grain quality. Grain chalkiness not only affects grain appearance, but also has adverse effects on milling and cooking performance. Chalkiness is a complex quantitative trait, which is easily affected by environments. In the early and middle stages of seed development, the occurrence of temperature stress will cause uneven seed filling and storage biosynthesis obstacles, leading to the formation of chalkiness (Masutomi et al. 2015; Sreenivasulu et al. 2015; Morita et al. 2016). The chalkiness of rice varieties varied greatly. It was found that the grain chalkiness of newly developed varieties was higher than that of old modern varieties, and that of the hybrid varieties was higher than that of other modern varieties (Laborte et al. 2015). Therefore, high-yielding varieties usually have higher chalkiness levels (Misra et al. 2019).

Percentage of grain chalkiness (PGC) is a quantitative index of grain chalkiness controlled by quantitative trait loci (QTLs) (Sreenivasulu et al. 2015; Misra et al. 2019). More than one hundred of QTLs controlling chalkiness have been reported in rice genome (Sreenivasulu et al. 2015; Yang et al. 2021). Many QTLs for chalkiness were detected on chromosomes 5, 6 and 8 in different populations and environments (He et al. 1999; Tan et al. 2000; Wan et al. 2005; Hao et al. 2009; Liu et al. 2011, 2012; Chen et al. 2011, 2016; Guo et al. 2011; Li et al. 2014; Peng et al. 2014; Zhao et al. 2015, 2016; Gao et al. 2016; Yun et al. 2016; Wang et al. 2017; Zhu et al. 2018a; Misra et al. 2019, 2020). On chromosome 8, the hot-spot region of the QTLs for chalkiness was located on the long arm (Li et al. 2003; Wan et al. 2005; Hao et al. 2009; Guo et al. 2011; Liu et al. 2012; Gao et al. 2016; Zhao et al. 2016; Wang et al. 2017). Although many chalk QTLs were reported in the hot-spot chromosome region, only one of the QTLs was usually detected in a population. Therefore, it is not clear how many chalk QTLs there are in the hot-spot chromosome region. It was found that the effect of QTLs for chalkiness is easily affected by high temperature. A set of QTLs for chalkiness were detected under heat stress condition (Kobayashi et al. 2007; Tabata et al. 2007; Wada et al. 2015; Miyahara et al. 2017; Nevame et al. 2018). Recently, we mapped two QTLs for grain chalkiness, *qPGC9* and *qPGC11*, and found that their additive effects on chalkiness significantly decreased under higher temperature (Yang et al. 2021).

The QTL effect of grain chalkiness was usually low, which was easily affected by other genetic factors. Several fine-mapped target genes and GWAS loci have been found to influence chalkiness, but many have either low or moderate effect (Gong et al. 2017; Wang et al. 2017; Quero et al. 2018; Misra et al. 2019). Although 11 GWAS loci for chalky grain rate were identified, the GWAS loci could only explain a small part of the phenotypic variation (Gong et al. 2017). Grain chalkiness and grain shape are two important traits for rice grain quality. It was found that grain width was positively correlated with chalkiness (Zhao et al. 2015). Some QTLs controlling grain width were overlapped with chalk QTLs (Wang et al. 2017). However, Misra et al. (2020) reported that only 3 of 78 QTL regions overlapped with known grain width genes, and the phenotypic variation for chalkiness showed a weak positive correlation with grain width. They believed that the key genes of grain width are not causal factors of chalkiness. Therefore, the genetic architecture of grain chalkiness and grain shape of rice remains unclear.

Substitution mapping is a powerful tool to detect QTLs for complex traits (Tan et al. 2021a, b; Yang et al. 2021). Like near-isogenic lines (NILs), single-segment substitution lines (SSSLs) carry only one substitution segment from donors in the recipient genetic background (Zhang et al. 2004; Keurentjes et al. 2007). We have developed a library of 2360 SSSLs, which were derived from 43 donors of 7 species of rice AA genome in the genetic background of Huajingxian 74 (HJX74), an *indica* elite variety in southern China (Zhang et al. 2004; Xi et al. 2006; Zhang 2019, 2021). These SSSLs were widely used to detect QTLs for complex traits, to clone QTLs of agronomic importance and to mine alleles of different functions (Zeng et al. 2006; Teng et al. 2012; Wang et al. 2012; Zhang et al. 2012; Zhu et al. 2014, 2018b; Yang et al. 2016, 2021; Zhou et al. 2017; Fang et al. 2019; Sui et al. 2019; Tan et al. 2020, 2021a, b). Recently, the SSSLs were used to detect QTLs controlling stigma exsertion rate (SER) of rice. Two pairs of tightly linked QTLs for SER, *qSER-2a* and *qSER-2b* on chromosome 2 and *qSER-3a* and *qSER-3b* on chromosome 3, were dissected by substitution mapping (Tan et al. 2021a). In previous study, we fine-mapped two QTLs *qPGC9* and *qPGC11* for grain chalkiness on rice chromosomes 9 and 11, which were sensitive to high temperature (Yang et al. 2021). In the present study, two closely linked QTLs for grain chalkiness on chromosome 8, *qPGC8.1* and *qPGC8.2*, were detected. The two QTLs affected on grain chalkiness and grain shape, and were insensitive to high temperature. Dissection of the two closely linked QTLs laid a foundation for revealing the genetic architecture of grain chalkiness in rice.

## Results

### Grain Chalkiness in SSSLs

**Fig. 1:**
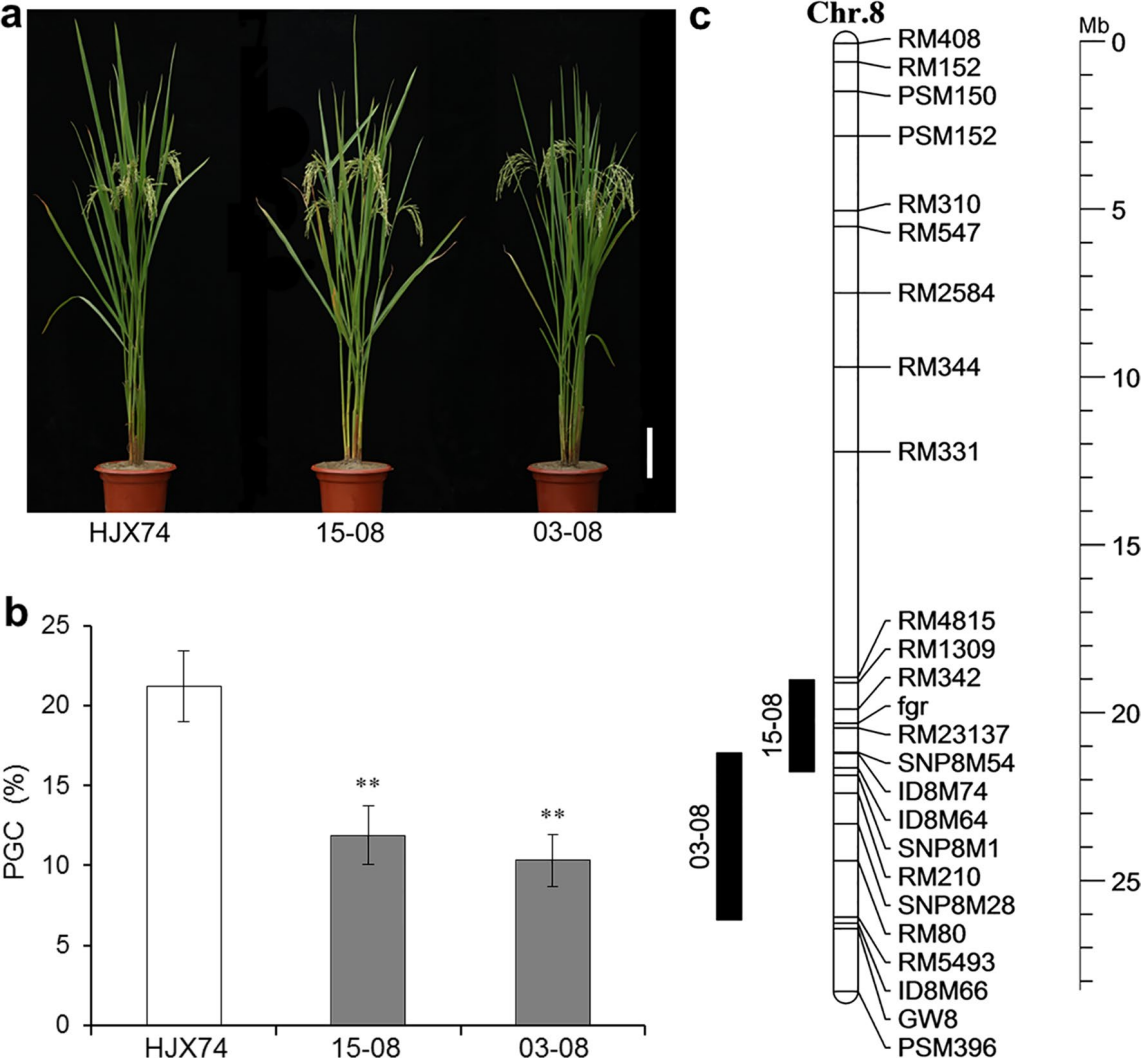
Grain chalkiness in HJX74 and SSSLs. **a** Plant types of HJX74 and SSSLs 15-08 and 03-08. Scale bar: 15 cm. **b** Percentage of grain chalkiness (PGC) (%) in HJX74 and SSSLs. PGC is the mean ± S.E. in six cropping seasons. **Represents the difference at 1% level of significance. **c** Chromosome locations of the two SSSLs. Physical distance (Mb) is shown as rulers on the right of chromosome. Black bars on the left of the chromosome 8 represent the estimated length of substitution segments in the SSSLs with their code on the left. The functional markers fgr and GW8 represent the loci of *fgr* gene for fragrance and *GW8* gene controlling grain width. *Chr.* chromosome, *Mb* megabase

Two SSSLs 15-08 and 03-08 with lower grain chalkiness were selected from the HJX74-SSSL library (Fig. 1a). The SSSLs were used to investigate grain chalkiness in consecutive 6 cropping seasons from the first cropping season (FCS) of 2017 to the second cropping season (SCS) of 2019. On average, the PGC of 15-08 and 03-08 was 11.9% and 10.3% respectively, which were significantly lower than 21.2% of recipient HJX74 (Fig. 1b and Additional file 1: Table S1). It meant that the two SSSLs each carried a QTL for PGC on their substitution segments.

Scanning electron microscopy (SEM) images showed contrasting differences in the microstructure of milled grains between HJX74 and SSSLs. The typical characteristics of chalkiness with the starch granules being small spherical and loosely packed in the opaque endosperm cells were observed in the grains with chalkiness in HJX74. By contrast, in the grains without chalkiness in SSSLs 15-08 and 03-08, compound starch granules were polyhedral and tightly packed, with no air spaces within or between them (Additional file 2: Fig. S1).

The substitution segments of 15-08 and 03-08 were surveyed by densifying molecular markers (Additional file 1: Table S2). The estimated length of substitution segments was 2726.7 kb in 15-08 and 4974.3 kb in 03-08. The two substitution segments overlapped in a 553.7 kb interval (Fig. 1c and Additional file 1: Table S3).

Eight agronomic traits of 15-08 and 03-08 were investigated. Most traits of the SSSLs were not significantly different from those of HJX74 (Fig. 1a and Additional file 1: Table S4). However, the grain shapes of 15-08 and 03-08 were significantly different from those of HJX74. For 15-08, the grain width was significantly narrower than that of HJX74, the former was 2.62 mm and 2.45 mm and the later was 2.69 mm and 2.64 mm in SCS of 2018 and in FCS of 2019, respectively. Compared with HJX74, 03-08 showed significantly longer in grain length and significantly narrower in grain width. In SCS of 2018 and in FCS of 2019, 03-08 had 9.14 mm and 8.88 mm in grain length and 2.55 mm and 2.40 mm in grain width, respectively (Additional file 1: Table S4). It is noted that *GW8* is outside the substitution segment of 03-08 (Fig. 1c). Therefore, the difference of grain shape between 03-08 and HJX74 was not controlled by *GW8* gene.

### Substitution Mapping of *qPGC8.1*

**Fig. 2:**
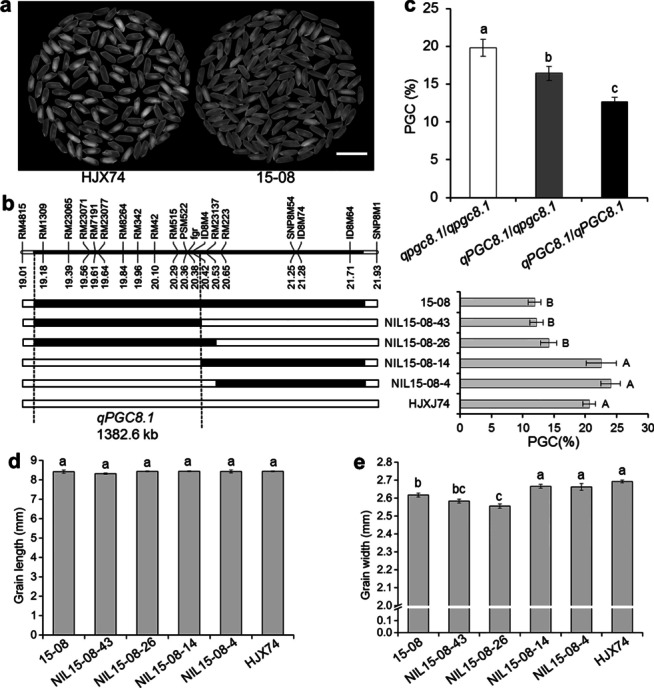
Substitution mapping of *qPGC8.1* for grain chalkiness*.* **a** The milled rice of the HJX74 and SSSL 15-08. Scale bar: 1 cm. **b** Substitution mapping of *qPGC8.1*. The positions of substitution segments and the percentage of grain chalkiness (PGC) of 15-08 and its NILs are shown. The numbers under the chromosome indicate physical distance (Mb). White and black blocks represent the homozygous genotypes of HJX74 and 15-08, respectively. PGC (%) was the mean ± S.E. in two cropping seasons. **c** PGC of three genotypes of *qPGC8.1* in an F_2_ population. *qpgc8.1*/*qpgc8.1* represents homozygous genotype of HJX74 (n = 24); *qPGC8.1*/*qpgc8.1* represents heterozygous genotype of HJX74/15-08 (n = 40); *qPGC8.1*/*qPGC8.1* represents homozygous genotype of 15-08 (n = 22). **d** Grain length of 15-08 and its NILs. **e** Grain width of 15-08 and its NILs. Values in the lines among different letters are different at 1% level of significance in **b** and at 5% level of significance in **c**–**e**

To map the QTL for grain chalkiness on the substitution segment of 15-08, the SSSL was used to develop secondary SSSLs or NILs. Four NILs were developed from an F_2:3_ population derived from the cross of HJX74/15-08. The four NILs were then investigated for grain chalkiness. PGC levels of two NILs, NIL15-08-26 and NIL15-08-43, were as low as 15-08, while those of other two NILs, NIL15-08-4 and NIL15-08-14, were as high as HJX74. Substitution segments of the two NILs with low PGC overlapped in the region between markers RM4815 and RM23137, while substitution segments of other two NILs with high PGC located outside the region. These results indicated that the QTL for grain chalkiness, *qPGC8.1*, was located in the region between markers RM4815 and RM23137 with the estimated interval length of 1382.6 kb (Fig. 2a, b).

Using RM8264 marker in *qPGC8.1* interval, Chi-square test was performed in 86 individuals of F_2_ population. The results showed that the segregation ratio of the three marker genotypes was 1:2:1 (χ^2^ = 0.51 < χ_0.01, 2_^2^ = 9.21). The effect of heterozygous genotype (*qPGC8.1*/*qpgc8.1*) was significantly higher than that of dominant homozygous genotype (*qPGC8.1*/*qPGC8.1*) and significantly lower than that of recessive homozygous genotype (*qpgc8.1*/*qpgc8.1*). The result showed that the effect of *qPGC8.1* on grain chalkiness was incomplete dominance (Fig. 2c).

Like 15-08, the four NILs showed no-significant difference from HJX74 in grain length (Fig. 2d). However, the grain width segregated in the NILs. Two NILs, NIL15-08-26 and NIL15-08-43, with low chalkiness showed narrower grain as 15-08, while other two NILs, NIL15-08-4 and NIL15-08-14, with high chalkiness were wider grain as HJX74, the former was 2.56 mm and 2.58 mm and the later was 2.66 mm and 2.67 mm, respectively (Fig. 2e). The results indicated that *qPGC8.1* controlled grain width besides grain chalkiness.

### Substitution Mapping of *qPGC8.2*

**Fig. 3:**
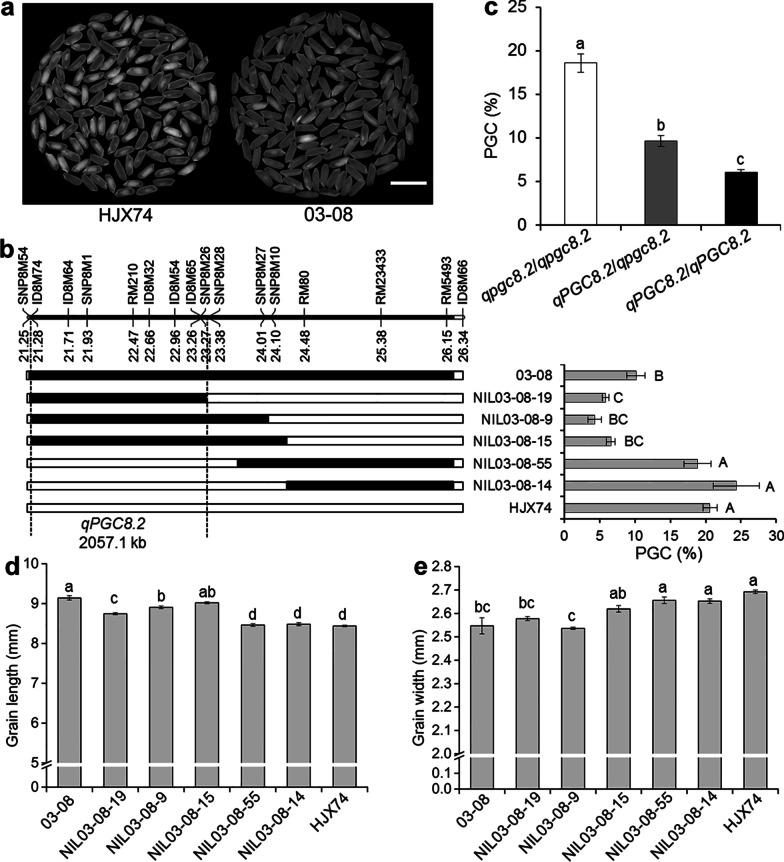
Substitution mapping of *qPGC8.2* for grain chalkiness*.* **a** The milled rice of the HJX74 and SSSL 03-08. Scale bar: 1 cm. **b** Substitution mapping of *qPGC8.2*. The positions of substitution segments and the percentage of grain chalkiness (PGC) of 03-08 and its NILs are shown. The numbers under the chromosome indicate physical distance (Mb). White and black blocks represent the homozygous genotypes of HJX74 and 03-08, respectively. PGC (%) was the mean ± S.E. in two cropping seasons. **c** PGC of three genotypes of *qPGC8.2* in an F_2_ population. *qpgc8.2*/*qpgc8.2* represents homozygous genotype of HJX74 (n = 23); *qPGC8.2*/*qpgc8.2* represents heterozygous genotype of HJX74/03-08 (n = 47); *qPGC8.2*/*qPGC8.2* represents homozygous genotype of 03-08 (n = 30). **d** Grain length of 03-08 and its NILs. **e** Grain width of 03-08 and its NILs. Values in the lines among different letters are different at 1% level of significance in **b** and at 5% level of significance in **c**–**e**

To map the QTL for grain chalkiness on the substitution segment of 03-08, the SSSL was used to develop secondary SSSLs or NILs. Five NILs were developed from an F_2:3_ population derived from the cross of HJX74/03-08. The five NILs were then investigated for grain chalkiness. PGC levels of three NILs, NIL03-08-9, NIL03-08-15 and NIL03-08-19, were as low as 03-08, while those of other two NILs, NIL03-08-14 and NIL03-08-55, were as high as HJX74. Substitution segments of the three NILs with low PGC overlapped in the region between markers SNP8M54 and SNP8M28, while substitution segments of other two NILs with high PGC located outside the region. These results indicated that the QTL for grain chalkiness, *qPGC8.2*, was located in the region between markers SNP8M54 and SNP8M28 with the estimated interval length of 2057.1 kb (Fig. 3a, b).

In the F_2_ population of 100 individuals, segregation ratio of three marker genotypes of RM210 in *qPGC8.2* region was 1:2:1 (χ^2^ = 1.34 < χ_0.01, 2_^2^ = 9.21). The effect of heterozygous genotype (*qPGC8.2*/*qpgc8.2*) was significantly different from that of the homozygous genotypes (*qPGC8.2*/*qPGC8.2* and *qpgc8.2*/*qpgc8.2*). The result showed that the effect of *qPGC8.2* on grain chalkiness was incomplete dominance (Fig. 3c).

In substitution mapping, NILs segregated in grain length and grain width. Three NILs, NIL03-08-9, NIL03-08-15 and NIL03-08-19, with low chalkiness showed longer and narrower grains as 03-08, while other two NILs, NIL03-08-14 and NIL03-08-55, with high chalkiness had shorter and wider grains as HJX74. The three NILs with low chalkiness were 8.75 mm, 8.91 mm and 9.02 mm in grain length and 2.58 mm, 2.54 mm and 2.62 mm in grain width, while the two NILs with high chalkiness were 8.46 mm and 8.48 mm in grain length and 2.66 mm and 2.65 mm in grain width, respectively (Fig. 3d, e). The results indicated that *qPGC8.2* controlled grain shape besides grain chalkiness.

It was noted that *qPGC8.2* was closely linked with *qPGC8.1* on chromosome 8. The maximum distance of the two QTL location was 4.37 Mb, from markers RM4815 to SNP8M28. The space distance between the two QTL regions was 0.72 Mb, from markers RM23137 to SNP8M54 (Figs. 1c, 2b, 3b).

### Influence of Different Cropping Seasons in Grain Chalkiness

The grain chalkiness was tested in two cropping seasons per year. During flowering to harvest of rice, the day and night temperatures of FCS and SCS were very different. In 2017–2019, the average values of maximum, minimum and mean temperatures were 32.1 °C, 25.9 °C and 29.0 °C in FCS, and 28.6 °C, 21.0 °C and 24.9 °C in SCS, respectively. The mean temperature in SCS was 4.1 °C lower than that in FCS (Additional file 1: Table S5).

**Fig. 4:**
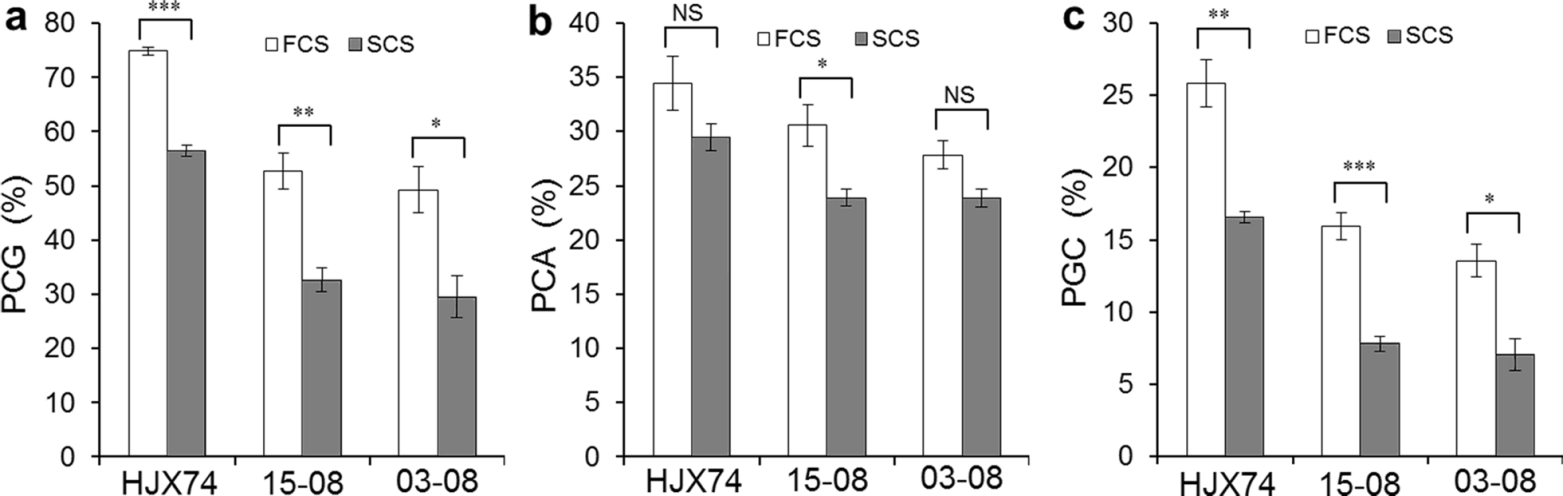
The difference of chalky traits between first cropping seasons (FCS) and second cropping seasons (SCS) in HJX74, 15-08and 03-08. **a** Percentage of chalky grain (PCG). **b** Percentage of chalky area (PCA). **c** Percentage of grain chalkiness (PGC). *, ** and ***indicate the difference at 0.05, 0.01 and 0.001 levels of significance, respectively. *NS* no significance

The grain chalkiness was lower in SCS than that in FCS in all lines. Percentage of chalky grains (PCG) and PGC were significantly different in all lines. Percentage of chalk area (PCA) was significantly different in 15-08, and no significantly different in 03-08 and HJX74. The PGC of HJX74, 15-08 and 03-08 was 25.8%, 15.9% and 13.6% in FCS, and 16.6%, 7.8% and 7.1% in SCS, respectively (Fig. 4). It is obvious that high temperature had great influence in PGC of HJX74 and SSSLs during seed filling period, mainly through the influence in PCG.

### Additive Effects of *qPGC8.1* and *qPGC8.2* on Grain Chalkiness

**Fig. 5:**
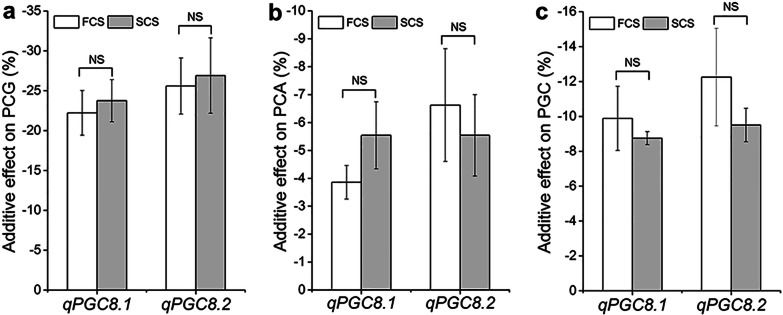
The additive effects of *qPGC8.1* and *qPGC8.2* on grain chalkiness in first cropping seasons (FCS) and second cropping seasons (SCS). **a** Percentage of chalky grain (PCG). **b** Percentage of chalky area (PCA). **c** Percentage of grain chalkiness (PGC). *NS* no significance

According to the estimation of chalkiness phenotype in 2017–2019, the additive effects of *qPGC8.1* and *qPGC8.2* had no significant difference between SCS and FCS. For *qPGC8.1*, the additive effects on PGC were −9.9% in FCS and –8.8% in SCS. For *qPGC8.2*, the additive effects on PGC in FCS and SCS were −12.3% and −9.5%, respectively (Fig. 5). The results showed that the additive effects of *qPGC8.1* and *qPGC8.2* didn't decrease in the high temperature condition of FCS. Therefore, the two QTLs were insensitive to high temperature.

## Discussion

### Dissection of Two Closely Linkage QTLs *qPGC8.1* and *qPGC8.2* on Chromosome 8

Grain chalkiness is a complex polygenic quantitative trait (Sreenivasulu et al. 2015). More than one hundred of QTLs for the chalkiness traits have been reported across all 12 chromosomes of rice genome (Sreenivasulu et al. 2015). Many QTLs for chalkiness in different populations were located on the long arm of chromosome 8. In the hot-spot region, however, only one chalk QTL was detected in each population (Li et al. 2003; Wan et al. 2005; Hao et al. 2009; Guo et al. 2011; Liu et al. 2012; Gao et al. 2016; Zhao et al. 2016; Wang et al. 2017). In the present study, we detected two closely linked QTLs, *qPGC8.1* and *qPGC8.2*, in the region of 19.01–23.38 Mb of chromosome 8. The maximum distance of the two QTL location was 4.37 Mb and the space distance between the two QTL regions was 0.72 Mb (Figs. 1c, 2b, 3b). Dissection of the two closely linked QTLs showed that the hot-spot region of chromosome 8 contained multiple QTLs for grain chalkiness. The results revealed more details of the genetic architecture of grain chalkiness in the hot-spot region of chromosome 8.

Recently, two pairs of tightly linked QTLs controlling SER, *qSER-2a* and *qSER-2b* on chromosome 2 and *qSER-3a* and *qSER-3b* on chromosome 3, were dissected by substitution mapping. On chromosome 2, two linkage QTLs, *qSER-2a* and *qSER-2b*, were located in the region of 1288.0 kb, and were respectively delimited to the intervals of 234.9 kb and 214.3 kb. On chromosome 3, two QTLs, *qSER-3a* and *qSER-3b*, were detected in the region of 3575.5 kb and were narrowed down to 319.1 kb and 637.3 kb, respectively (Tan et al. 2021a). Together, those results indicated that substitution mapping using SSSLs is a powerful tool for dissection of closely linked QTLs for complex traits.

### Relationship Between Grain Chalkiness and Grain Shape During Grain Development

Grain chalkiness and grain shape are two important traits for rice grain quality. It was found that grain width had a negative pleiotropic effect on grain chalkiness (Zhao et al. 2015; Gao et al. 2016). *GW2* allele significantly increased grain weight and grain width, but also led to increase chalkiness in rice (Song et al. 2007). The *gw8*, *GW7* and *gs9* alleles exhibited more slender grain phenotype and reduced chalkiness, which significantly improved the appearance quality of rice (Wang et al. 2012, 2015; Zhao et al. 2018). Zhao et al. (2015) identified three QTLs for chalkiness tightly linked to *GS3*, *gw5*, and *qGL7-2* controlling grain size. However, grain chalkiness was weakly positively correlated with phenotypic variation of grain width, and the key genes affecting grain width were not the cause of chalkiness (Misra et al. 2020). Recently, we detected two QTLs for grain chalkiness, *qPGC9* and *qPGC11*, by substitution mapping, and found that they had no effect on grain shape (Yang et al. 2021). Therefore, the relationship between grain chalkiness and grain shape in rice remains unclear. In the present study, two closely linked QTLs, *qPGC8.1* and *qPGC8.2*, on chromosome 8 showed effects on grain chalkiness and grain shape (Figs. 2, 3). In addition, it is noted that the *fgr* gene for fragrance (Bradbury et al. 2005) is located not only on the substitution segment of 15-08 but also in the *qPGC8.1* mapped region, and the *GW8* gene controlling grain width (Wang et al. 2012) is located near the *qPGC8.2* mapped region although it is outside the substitution segment of 03-08 (Fig. 1c). These results indicated that the long arm of chromosome 8 may be a gene cluster area for grain development. It is unclear that each region of *qPGC8.1* and *qPGC8.2* carries two different genes, which control grain chalkiness and grain shape respectively, or one gene that controls grain chalkiness and has a pleiotropic effect on grain shape. Therefore, the mapping of *qPGC8.1* and *qPGC8.2* laid a foundation for revealing the relationship between chalkiness and grain shape during grain development.

### Influence of High Temperature in the Effect of Chalk QTLs

It was found that the high temperature during the seed filling stage caused uneven grain filling and resulted in chalk formation (Sreenivasulu et al. 2015; Masutomi et al. 2015; Morita et al. 2016; Ishimaru et al. 2019). In Guandong province of China, rice is planted in two cropping seasons per year. During the seed filling period, the air temperature of FCS is usually higher than that of SCS. In 2017–2019, the mean temperature of FCS was 4.1 °C higher than that of SCS (Additional file 1: Table S5). Due to higher temperature, grain chalkiness of all lines in FCS was significantly higher than that in SCS (Fig. 4). Obviously, the grain chalkiness of HJX74 and SSSLs was greatly affected by higher temperature during the seed filling period.

Some QTLs for chalkiness were detected under high temperature stress (Nevame et al. 2018). Kobayashi et al. (2007) detected three QTLs for chalkiness in *japonica* varieties under heat stress. Tabata et al. (2007) identified four QTLs for chalkiness in a RIL population derived from a cross between a heat stress-tolerant variety and a heat stress-sensitive variety. Wada et al. (2015) and Miyahara et al. (2017) identified a set of chalk QTLs under heat stress condition using a RIL population derived from a cross between a heat-tolerant variety and a heat stress-sensitive variety. Recently, we mapped two QTLs for grain chalkiness, *qPGC9* and *qPGC11*, and found that the additive effects of *qPGC9* and *qPGC11* on chalkiness in SCS were almost twice of those in FCS. The additive effects of *qPGC9* and *qPGC11* on chalkiness greatly decreased by high temperature of FCS. Therefore, the *qPGC9* and *qPGC11* were sensitive to high temperature (Yang et al. 2021). These results showed that many QTLs could increase chalkiness under high temperature. This may be the reason why most varieties show high chalkiness at high temperature. In the present study, the grain chalkiness of HJX74 and SSSLs was greatly affected by higher temperature during the seed filling period. It is because they have the same genetic background carrying some QTLs increasing chalkiness under high temperature. Interestingly, the additive effects of *qPGC8.1* and *qPGC8.2* on PCG, PCA and PGC were no significant difference in different cropping seasons (Fig. 5), showing that the additive effects of *qPGC8.1* and *qPGC8.2* on grain chalkiness were not affected by the high temperature in FCS. It indicates that *qPGC8.1* and *qPGC8.2* are insensitive to high temperature, which are different from *qPGC9* and *qPGC11*. Therefore, *qPGC8.1* and *qPGC8.2* can be used for the breeding of rice varieties with lower grain chalkiness under high temperature.

## Conclusion

Two closely linked QTLs *qPGC8.1* and *qPGC8.2* controlling grain chalkiness and grain shape were located on chromosome 8. The effect of *qPGC8.1* and *qPGC8.2* was incomplete dominance. The additive effects of the two QTLs on grain chalkiness had no significant difference between FCS and SCS. The *qPGC8.1* and *qPGC8.2* were insensitive to the high temperature in FCS. The mapping of *qPGC8.1* and *qPGC8.2* laid a foundation for revealing the relationship between grain chalkiness and grain shape during grain development. The two QTLs are favorable for the breeding of rice varieties with low chalkiness under high temperature condition.

## Materials and methods

### Rice Materials and Cropping Seasons

Two SSSLs 03-08 and 15-08 with lower grain chalkiness were selected from the HJX74-SSSL library. The substitution segment of 03-08 was from the donor Zhong4188 and that of 15-08 was from the donor American Jasmine. The donors of both SSSLs are *indica* varieties. All rice materials were planted at the farm of South China Agricultural University, Guangzhou (23°07′N, 113°15′E) from 2017 to 2019. The materials were planted in two cropping seasons per year, the FCS from late February to middle July and the SCS from late July to middle November. Rice cultivation and controlling of diseases and insect pests were common practices in southern China.

### Measurement of Grain Chalkiness

The seeds of each line were harvested after full maturity. The dried seeds of 10 plants of each line were separately processed into milled rice, and 200 head rice grains of each plant were randomly selected for measurement of chalkiness (Yang et al. 2021). Images of head rice grains were captured and the chalkiness parameters were measured by Microtek ScanWizard EZ scanner and rice quality analyzer SC-E software (Hangzhou Wanshen Detection Technology Co., Ltd., Hangzhou, China, www.wseen.com). PGC is the product of PCG in total grains multiplied by PCA per chalky grain. Endosperm microstructure was observed by SEM. Milled grains were transversely cut with a razor blade. The thin and clean fracture was fixed on SEM stubs and sputter-coated with gold under vacuum. The fixed specimens were observed under a scanning electron microscope (ZEISS EVO MA 15).

### Phenotyping of Traits and Statistical Analysis

Heading date, plant height and panicle number per plant was investigated in the field. Grain traits were measured by the yield traits scorer (YTS), a rice phenotypic facility (Yang et al. 2014). The percentages were converted to the arcsine square root for statistical analysis. The student’s *t*-test was used for comparison between two groups. Dunnett *t*-test was used to compare multiple groups with control group. Least significance range (LSR) was used for multiple range test among multiple groups (Duncan 1955). The data analysis and figure making were done by SPSS statistics 23.0 and OriginPro 9.0 (https://www.originlab.com).

### Genotyping of Molecular Markers and Substitution Mapping

Molecular markers labeled “RM” were selected from online resources (https://archive.gramene.org/markers/). New markers used in this study were designed using the software of Primer Premier 5.0 (Lalitha 2000). The DNA samples were amplified by PCR method. The PCR products were separated by gel electrophoresis on 6% denatured PAGE and detected by the silver staining method (Tan et al. 2020). To develop secondary SSSLs or NILs, 03-08 and 15-08 were crossed with the recipient HJX74. The NILs were developed from F_2:3_ populations derived from the crosses. Minimum length, maximum length and estimated length of a substitution segment were estimated as described by Tan et al. (2020). When PGC showed significant difference between SSSL genotype and HJX74 genotype, a QTL for PGC was detected on the substitution segment of SSSL. When multiple NILs showed significantly different phenotype from HJX74 and their substitution segments overlapped, the QTL was located in the overlapping region (Eshed and Zamir, 1995; Tan et al. 2020). Additive effect of a QTL was defined as the phenotypic difference between SSSL and HJX74 (Zhou et al. 2020). The naming of QTLs followed the rules proposed by McCouch et al. (1997).

## Supplementary Information

**Additional file 1: Table S1**. Phenotypes of chalky traits in SSSLs. **Table S2**. Markers developed to detect the substitution segments of SSSLs. **Table S3**. Substitution segments of SSSLs. **Table S4**. Phenotypes of agronomic traits in SSSLs. **Table S5**. Average temperatures for 30 days after flowering of rice in two cropping seasons.

**Additional file 2: Fig. S1**. Scanning electron microscopy (SEM) images of endosperm transverse sections from milled grains. In the grain with chalkiness in HJX74 (a), the starch granules loosely pack in the opaque endosperm cells. In the grains without chalkiness in SSSLs 15-08 (b) and 03-08 (c), compound starch granules are tightly packed, with no air spaces within or between them. Scale bar: 10 μm.

## Data Availability

All data generated or analyzed in this study are included in this published article and its additional information files.

## Abbreviations

FCS: First cropping season
HJX74: Huajingxian 74
NIL: Near-isogenic line
PCA: Percentage of chalky area
PCG: Percentage of chalky grain
PGC: Percentage of grain chalkiness
QTL: Quantitative trait locus
SCS: Second cropping season
SEM: Scanning electron microscopy
SER: Stigma exsertion rate
SSSL: Single-segment substitution line

## References

[CR1] Bradbury LMT, Fitzgerald TL, Henry RJ, Jin Q, Waters DLE (2005). The gene for fragrance in rice. Plant Biotechnol J.

[CR2] Chen H, Zhao Z, Jiang L, Wan X, Liu L, Wu X, Wan J (2011). Molecular genetic analysis on percentage of grains with chalkiness in rice (*Oryza sativa* L.). Afr J Biotechnol.

[CR3] Chen L, Gao W, Chen S, Wang L, Zou J, Liu Y, Wang H, Chen Z, Guo T (2016). High-resolution QTL mapping for grain appearance traits and co-localization of chalkiness-associated differentially expressed candidate genes in rice. Rice.

[CR4] Duncan DB (1955). Multiple range and multiple *F* tests. Biometrics.

[CR5] Eshed Y, Zamir D (1995). An introgression line population of *Lycopersicon pennellii* in the cultivated tomato enables the identification and fine mapping of yield-associated QTL. Genetics.

[CR6] Fang C, Li L, He R, Wang D, Wang M, Hu Q, Ma Q, Qin K, Feng X, Zhang G, Fu X, Liu Z (2019). Identification of *S23* causing both interspecific hybrid male sterility and environment-conditioned male sterility in rice. Rice.

[CR7] Gao Y, Liu C, Li Y, Zhang A, Dong G, Xie L, Zhang B, Ruan B, Hong K, Xue D, Zeng D, Guo L, Qian Q, Gao Z (2016). QTL analysis for chalkiness of rice and fine mapping of a candidate gene for *qACE9*. Rice.

[CR8] Gong J, Miao J, Zhao Y, Zhao Q, Feng Q, Zhan Q, Cheng B, Xia J, Huang X, Yang S, Han B (2017). Dissecting the genetic basis of grain shape and chalkiness traits in hybrid rice using multiple collaborative populations. Mol Plant.

[CR9] Guo T, Liu X, Wan X, Weng J, Liu S, Liu X, Chen M, Li J, Su N, Wu F, Cheng Z, Guo X, Lei C, Wang J, Jiang L, Wan J (2011). Identification of a stable quantitative trait locus for percentage grains with white chalkiness in rice (*Oryza sativa*). J Integr Plant Biol.

[CR10] Hao W, Zhu M, Gao J, Sun S, Lin H (2009). Identification of quantitative trait loci for rice quality in a population of chromosome segment substitution lines. J Integr Plant Biol.

[CR11] He P, Li SG, Qian Q, Ma YQ, Li JZ, Wang WM, Chen Y, Zhu LH (1999). Genetic analysis of rice grain quality. Theor Appl Genet.

[CR12] Ishimaru T, Parween S, Saito Y, Shigemitsu T, Yamakawa H, Nakazono M, Masumura T, Nishizawa NK, Kondo M, Sreenivasulu N (2019). Laser microdissection-based tissue-specific transcriptome analysis reveals a novel regulatory network of genes involved in heat-induced grain chalk in rice endosperm. Plant Cell Physiol.

[CR13] Keurentjes JJB, Bentsink L, Alonso-Blanco C, Hanhart CJ, Vries HBD, Effgen S, Vreugdenhil D, Koornneef M (2007). Development of a near-isogenic line population of *Arabidopsis thaliana* and comparison of mapping power with a recombinant inbred line population. Genetics.

[CR14] Kobayashi A, Genliang B, Shenghai Y, Tomita K (2007). Detection of quantitative trait loci for white-back and basal-white kernels under high temperature stress in *japonica* rice varieties. Breed Sci.

[CR15] Laborte AG, Paguirigan NC, Moya PF, Nelson A, Sparks AH, Gregorio GB (2015). Farmers’ preference for rice traits: insights from farm surveys in central Luzon, Philippines, 1966–2012. PLoS ONE.

[CR16] Lalitha S (2000). Primer premier 5. Biotech Softw Internet Rep.

[CR17] Li ZF, Wan JM, Xia JF, Zhai HQ (2003). Mapping quantitative trait loci underlying appearance quality of rice grains (*Oryza sativa* L.). Acta Genet Sin.

[CR18] Li Y, Fan C, Xing Y, Yun P, Luo L, Yan B, Peng B, Xie W, Wang G, Li X, Xiao J, Xu C, He Y (2014). *Chalk5* encodes a vacuolar H^+^-translocating pyrophosphatase influencing grain chalkiness in rice. Nat Genet.

[CR19] Liu X, Wan X, Ma X, Wan J (2011). Dissecting the genetic basis for the effect of rice chalkiness, amylose content, protein content, and rapid viscosity analyzer profile characteristics on the eating quality of cooked rice using the chromosome segment substitution line population across eight environments. Genome.

[CR20] Liu X, Wang Y, Wang SW (2012). QTL analysis of percentage of grains with chalkiness in *Japonica* rice (*Oryza sativa*). Genet Mol Res.

[CR21] Masutomi Y, Arakawa M, Minoda T, Yonekura T, Shimada T (2015). Critical air temperature and sensitivity of the incidence of chalky rice kernels for the rice cultivar “sai-no-kagayaki”. Agr For Meteorol.

[CR22] McCouch SR, Cho YG, Yano M, Paul E, Blinstrub M, Mor-ishima H, Kinosita T (1997). II. Report from coordinators. (1) Report on QTL nomenclature. Rice Genet Newsl.

[CR23] Misra G, Anacleto R, Badoni S, Butardo V, Molina L, Graner A, Demont M, Morell MK, Sreenivasulu N (2019). Dissecting the genome-wide genetic variants of milling and appearance quality traits in rice. J Exp Bot.

[CR24] Misra G, Badoni S, Parween S, Singh RK, Leung H, Ladejobi O, Mott R, Sreenivasulu N (2020). Genome-wide association coupled gene to gene interaction studies unveil novel epistatic targets among major effect loci impacting rice grain chalkiness. Plant Biotechnol J.

[CR25] Miyahara K, Wada T, Sonoda J, Tsukaguchi T, Miyazaki M, Tsubone M, Yamaguchi O, Ishibashi M, Iwasawa N, Umemoto T, Kondo M (2017). Detection and validation of QTLs for milky-white grains caused by high temperature during the ripening period in *Japonica* rice. Breed Sci.

[CR26] Morita S, Wada H, Matsue Y (2016). Countermeasures for heat damage in rice grain quality under climate change. Plant Prod Sci.

[CR27] Nevame AYM, Emon RM, Malek MA, Hasan MM, Alam MA, Muharam FM, Aslani F, Rafii MY, Ismail MR (2018). Relationship between high temperature and formation of chalkiness and their effects on quality of rice. Biomed Res Int.

[CR28] Peng B, Wang L, Fan C, Jiang G, Luo L, Li Y, He Y (2014). Comparative mapping of chalkiness components in rice using five populations across two environments. BMC Genet.

[CR29] Quero G, Gutiérrez L, Monteverde E, Blanco P, Pérez De Vida F, Rosas J, Fernández S, Garaycochea S, McCouch S, Berberian N, Simondi S, Bonnecarrère V (2018). Genome-wide association study using historical breeding populations discovers genomic regions involved in high-quality rice. Plant Genome.

[CR30] Song X, Huang W, Shi M, Zhu M, Lin H (2007). A QTL for rice grain width and weight encodes a previously unknown RING-type E3 ubiquitin ligase. Nat Genet.

[CR31] Sreenivasulu N, Butardo VM, Misra G, Cuevas RP, Anacleto R, Kavi Kishor PB (2015). Designing climate-resilient rice with ideal grain quality suited for high-temperature stress. J Exp Bot.

[CR32] Sui F, Zhao D, Zhu H, Gong Y, Tang Z, Huang X, Zhang G, Zhao F (2019). Map-based cloning of a new total loss-of-function allele of *OsHMA3* causes high cadmium accumulation in rice grain. J Exp Bot.

[CR33] Tabata M, Hirabayashi H, Takeuchi Y, Ando I, Iida Y, Ohsawa R (2007). Mapping of quantitative trait loci for the occurrence of white-back kernels associated with high temperatures during the ripening period of rice (*Oryza sativa* L.). Breed Sci.

[CR34] Tan YF, Xing YZ, Li JX, Yu SB, Xu CG, Zhang Q (2000). Genetic bases of appearance quality of rice grains in Shanyou 63, an elite rice hybrid. Theor Appl Genet.

[CR35] Tan Q, Zou T, Zheng M, Ni Y, Luan X, Li X, Yang W, Yang Z, Zhu H, Zeng R, Liu G, Wang S, Fu X, Zhang G (2020). Substitution mapping of the major quantitative trait loci controlling stigma exsertion rate from *Oryza glumaepatula*. Rice.

[CR36] Tan Q, Wang C, Luan X, Zheng L, Ni Y, Yang W, Yang Z, Zhu H, Zeng R, Liu G, Wang S, Zhang G (2021). Dissection of closely linked QTLs controlling stigma exsertion rate in rice by substitution mapping. Theor Appl Genet.

[CR37] Tan Q, Zhu H, Liu H, Ni Y, Wu S, Luan X, Liu J, Yang W, Yang Z, Zeng R, Liu G, Wang S, Zhang G (2021b) Fine mapping of QTLs for stigma exsertion rate from *Oryza glaberrima* by chromosome segment substitution. Rice Sci 28, online at http://www.ricescience.org/fileup/PDF/2021-0003C.pdf

[CR38] Teng B, Zeng R, Wang Y, Liu Z, Zhang Z, Zhu H, Ding X, Li W, Zhang G (2012). Detection of allelic variation at the *Wx* locus with single-segment substitution lines in rice (*Oryza sativa* L.). Mol Breed.

[CR39] Wada T, Miyahara K, Sonoda J, Tsukaguchi T, Miyazaki M, Tsubone M, Ando T, Ebana K, Yamamoto T, Iwasawa N, Umemoto T, Kondo M, Yano M (2015). Detection of QTLs for white-back and basal-white grains caused by high temperature during ripening period in *japonica* rice. Breed Sci.

[CR40] Wan XY, Wan JM, Weng JF, Jiang L, Bi JC, Wang CM, Zhai HQ (2005). Stability of QTLs for rice grain dimension and endosperm chalkiness characteristics across eight environments. Theor Appl Genet.

[CR41] Wang S, Wu K, Yuan Q, Liu X, Liu Z, Lin X, Zeng R, Zhu H, Dong G, Qian Q, Zhang G, Fu X (2012). Control of grain size, shape and quality by *OsSPL16* in rice. Nat Genet.

[CR42] Wang S, Li S, Liu Q, Wu K, Zhang J, Wang S, Wang Y, Chen X, Zhang Y, Gao C, Wang F, Huang H, Fu X (2015). The *OsSPL16*-*GW7* regulatory module determines grain shape and simultaneously improves rice yield and grain quality. Nat Genet.

[CR43] Wang X, Pang Y, Wang C, Chen K, Zhu Y, Shen C, Ali J, Xu J, Li Z (2017). New candidate genes affecting rice grain appearance and milling quality detected by genome-wide and gene-based association analyses. Front Plant Sci.

[CR44] Xi Z, He F, Zeng R, Zhang Z, Ding X, Li W, Zhang G (2006). Development of a wide population of chromosome single-segment substitution lines in the genetic background of an elite cultivar of rice (*Oryza sativa* L.). Genome.

[CR45] Yang W, Guo Z, Huang C, Duan L, Chen G, Jiang N, Fang W, Feng H, Xie W, Lian X, Wang G, Luo Q, Zhang Q, Liu Q, Xiong L (2014). Combining high-throughput phenotyping and genome-wide association studies to reveal natural genetic variation in rice. Nat Commun.

[CR46] Yang T, Zhang S, Zhao J, Liu Q, Huang Z, Mao X, Dong J, Wang X, Zhang G, Liu B (2016). Identification and pyramiding of QTLs for cold tolerance at the bud bursting and the seedling stages by use of single segment substitution lines in rice (*Oryza sativa* L.). Mol Breed.

[CR47] Yang W, Liang J, Hao Q, Luan X, Tan Q, Lin S, Zhu H, Liu G, Liu Z, Bu S, Wang S, Zhang G (2021). Fine mapping of two grain chalkiness QTLs sensitive to high temperature in rice. Rice.

[CR48] Yun P, Zhu Y, Wu B, Gao G, Sun P, Zhang Q, He Y (2016). Genetic mapping and confirmation of quantitative trait loci for grain chalkiness in rice. Mol Breed.

[CR49] Zeng R, Zhang Z, He F, Xi Z, Talukdar A, Shi J, Qin L, Huang C, Zhang G (2006). Identification of multiple alleles at the *Wx* locus and development of single segment substitution lines for the alleles in rice. Rice Sci.

[CR50] Zhang G (2019) The platform of breeding by design based on the SSSL library in rice. Hereditas (Beijing) 41:754–760 (**in Chinese with English abstract**) 10.16288/j.yczz.19-105 31447426

[CR51] Zhang G (2021). Target chromosome segment substitution: a way to breeding by design in rice. Crop J.

[CR52] Zhang G, Zeng R, Zhang Z, Ding X, Li W, Liu G, He F, Tulukdar A, Huang C, Xi Z, Qin L, Shi J, Zhao F, Feng M, Shan Z, Chen L, Guo X, Zhu H, Lu Y (2004). The construction of a library of single segment substitution lines in rice (*Oryza sativa* L.). Rice Genet Newsl.

[CR53] Zhang Y, Yang J, Shan Z, Chen S, Qiao W, Zhu X, Xie Q, Zhu H, Zhang Z, Zeng R, Ding X, Zhang G (2012). Substitution mapping of QTLs for blast resistance with SSSLs in rice (*Oryza sativa* L.). Euphytica.

[CR54] Zhao X, Zhou L, Ponce K, Ye G (2015). The usefulness of known genes/QTLs for grain quality traits in an *indica* population of diverse breeding lines tested using association analysis. Rice.

[CR55] Zhao X, Daygon VD, McNally KL, Hamilton RS, Xie F, Reinke RF, Fitzgerald MA (2016). Identification of stable QTLs causing chalk in rice grains in nine environments. Theor Appl Genet.

[CR56] Zhao D, Li Q, Zhang C, Zhang C, Yang Q, Pan L, Ren X, Lu J, Gu M, Liu Q (2018). *GS9* acts as a transcriptional activator to regulate rice grain shape and appearance quality. Nat Commun.

[CR57] Zhou Y, Xie Y, Cai J, Liu C, Zhu H, Jiang R, Zhong Y, Zhang G, Tan B, Liu G, Fu X, Liu Z, Wang S, Zhang G, Zeng R (2017). Substitution mapping of QTLs controlling seed dormancy using single segment substitution lines derived from multiple cultivated rice donors in seven cropping seasons. Theor Appl Genet.

[CR58] Zhou H, Yang W, Ma S, Luan X, Zhu H, Wang A, Huang C, Rong B, Dong S, Meng L, Wang S, Zhang G, Liu G (2020). Unconditional and conditional analysis of epistasis between tillering QTLs based on single segment substitution lines in rice. Sci Rep.

[CR59] Zhu Y, Zuo S, Chen Z, Chen X, Li G, Zhang Y, Zhang G, Pan X (2014). Identification of two major rice sheath blight resistance QTLs, *qSB1-1*^HJX74^ and *qSB11*^HJX74^, in field trials using chromosome segment substitution lines. Plant Dis.

[CR60] Zhu A, Zhang Y, Zhang Z, Wang B, Xue P, Cao Y, Chen Y, Li Z, Liu Q, Cheng S, Cao L (2018a) Genetic dissection of *qPCG1* for a quantitative trait locus for percentage of chalky grain in rice (*Oryza sativa* L.). Front Plant Sci 9:1173 10.3389/fpls.2018.01173 PMC6095994 30147703

[CR61] Zhu H, Li Y, Liang J, Luan X, Xu P, Wang S, Zhang G, Liu G (2018b) Analysis of QTLs on heading date based on single segment substitution lines in rice (*Oryza sativa* L.). Sci Rep 8:13232 10.1038/s41598-018-31377-7 PMC6125461 30185925

