# Supplementary material for: Substitution Mapping of Two Closely Linked QTLs on Chromosome 8 Controlling Grain Chalkiness in Rice
Source: Rice (N Y). 2021 Oct 2;14:85. doi: 10.1186/s12284-021-00526-4 (PMC8487414; doi:10.1186/s12284-021-00526-4)
Supplement: Supplementary file 2 — Additional file 2: Fig. S1. Scanning electron microscopy (SEM) images of endosperm transverse sections from milled grains. In the grain with chalkiness in HJX74 (a), the starch granules loosely pack in the opaque endosperm cells. In the grains without chalkiness in SSSLs 15-08 (b) and 03-08 (c), compound starch granules are tightly packed, with no air spaces within or between them. Scale bar: 10 μm. [file 12284_2021_526_MOESM2_ESM.docx]

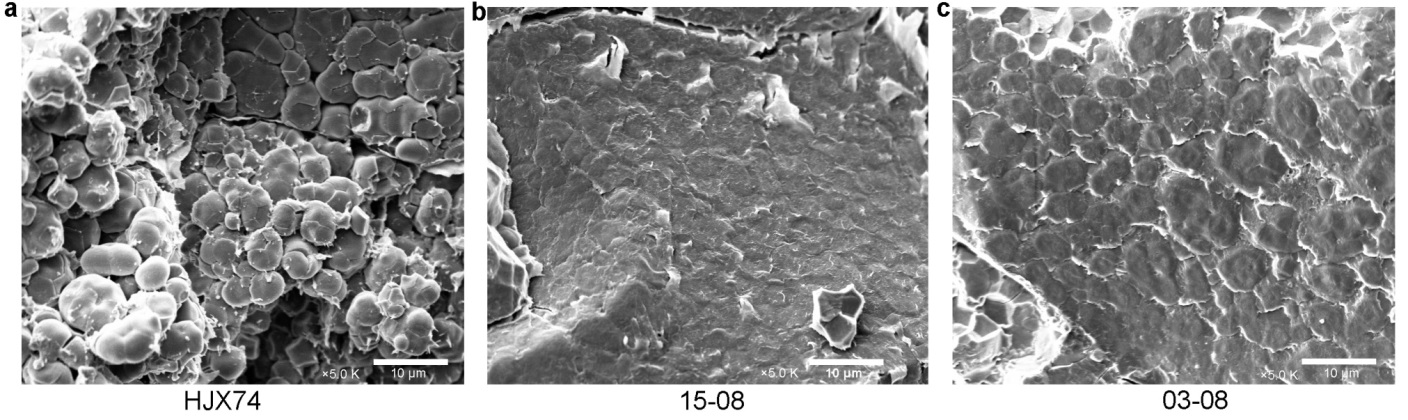
**Additional file 2**

Fig. S1 Scanning electron microscopy (SEM) images of endosperm transverse sections from milled grains. In the grain with chalkiness in HJX74 (a), the starch granules loosely pack in the opaque endosperm cells. In the grains without chalkiness in SSSLs 15-08 (b) and 03-08 (c), compound starch granules are tightly packed, with no air spaces within or between them. Scale bar: 10 μm.
